# Are Maternal Vitamin D (25(OH)D) Levels a Predisposing Risk Factor for Neonatal Growth? A Cross-Sectional Study

**DOI:** 10.3390/clinpract14010021

**Published:** 2024-02-05

**Authors:** Artemisia Kokkinari, Maria Dagla, Evangelia Antoniou, Aikaterini Lykeridou, Georgios Iatrakis

**Affiliations:** Department of Midwifery, School of Health & Care Sciences, University of West Attica, 12243 Athens, Greece; mariadagla@uniwa.gr (M.D.); lilanton@uniwa.gr (E.A.); klyker@uniwa.gr (A.L.); giatrakis@uniwa.gr (G.I.)

**Keywords:** pregnancy, maternal 25(OH)D concentrations, neonatal 25(OH)D concentrations, fetal blood, vitamin D deficiency, somatometric characteristics

## Abstract

Background: Neonatal bone mass may potentially be influenced by existing maternal vitamin D (25(OH)D) levels. Few studies evaluated maternal vitamin D deficiency (VDD) with neonatal anthropometrics such as weight, height and head circumference (HC), especially in Greece, which is a Mediterranean country with plenty of sunshine and consequently benefits the synthesis of 25(OH)D. We investigated this potential association in Greece, taking into account the administration or not of prenatal vitamin D supplements. The purpose of our study is to ascertain if there is a possible association between maternal VDD and neonatal specific anthropometric characteristics (weight, height and HC) at birth. If this is confirmed by future clinical studies, it would be of interest to develop a prenatal pregnancy selection program that would detect VDD early or during pregnancy in order to improve fetal–neonatal development in a Mediterranean country like ours. Methods: We performed a cross-sectional study on 248 early early term infants (after 37 + 0 to 38 + 6 weeks of gestation) but also on full-term infants (after 39 to 40 weeks of gestation) and their Greek mothers from September 2019 to January 2022. Blood samples of 25(OH)D were taken from the mother at the beginning of labor and cord blood was taken from the newborn. Pregnant women were divided into two groups: those who received or did not receive a normal dose of calcium (500 mg/day) and vitamin D supplements (400–800 IU/day) as instructed by their treating physicians. Results: Our findings revealed a positive association between maternal VDD and low neonate birth weight (LBW) in women receiving vitamin D during pregnancy and no association between maternal VDD and neonatal height or head circumference (HC) at birth. Conclusions: Overall, this study highlighted the association between maternal VDD at the end of gestation and LBW neonates born to mothers who received vitamin D supplementation. We did not find any correlation in two of the three somatometric characteristics studied, height and HC. In any case, more clinical studies are needed to further corroborate any potential association of maternal VDD with other neonatal somatometric characteristics.

## 1. Introduction

Vitamin D (25(OH)D) requirements during pregnancy are greater, as evidenced by the physiologically higher levels of 1,25(OH)2D, the major circulating form of vitamin D, seen in the second and the third trimesters of pregnancy [1]. We know that pregnancy modifies maternal levels of 25(OH)D and maternal calcium, with the ultimate goal of fetal calcium homeostasis. When maternal levels of 25(OH)D are insufficient, levels of maternal parathyroid hormone (PTH) increase to stimulate the bone absorption of fetal calcium and maintain its levels so as to cover the developing fetus [2]. Maintaining calcium concentrations in pregnancy is vital, as it appears to play an important role in fetal bone development. However, the effect of vitamin D supplementation on fetal bone development during pregnancy is unclear [3]. Although most observational studies recognize the important role of vitamin D in pregnancy and the possibility that vitamin D deficiency (VDD) is responsible for the unwanted complications of pregnancy, both in the mother and in the newborn [4], more clinical studies are needed, which may indicate the role of maternal 25(OH)D levels in the anthropometric characteristics of the newborn.

The results of a Greek study by Papadakis et al. [5] showed a seasonal variance of serum 25(OH)D concentrations in Athens, Greece, 37.9° north (N) of the Equator. Given this condition, the lowest 25(OH)D levels in serum concentrations are observed during the winter and early spring months. Greece extends between latitudes 34° and 42°, and the climate is typical of the Mediterranean region, with mild and rainy winters, relatively hot and dry summers and abundant sunshine during most of the year (Hellenic National Meteorological Service/HNMS) [6]. From a climatic point of view, the year can be divided into two main seasons: the cold and rainy season that lasts from mid-October to the end of March and the hot and dry season that lasts from April to October. Precipitation, even during the winter season, does not last for many days, and neither does the sky remain cloudy for long. Winter storms often cease in January or on the first days of February at the latest and are succeeded by sunny days, known since ancient times as the Halcyon Days. Therefore, the weak effect of sunlight, for the given latitude of Athens, could be a possible explanation for why serum 25(OH)D levels are higher in February compared to January and March. Full cloud cover reduces UV energy by 50%. Shade, including that from severe pollution, reduces UV energy by 60% (Hellenic National Meteorological Service/HNMS) [6]. Hosseinpanah et al. [7], who tried to determine whether air pollution and low ground levels of UVB light (UVB; 290–315) can deteriorate the body’s vitamin D status in healthy women, saw that living in a polluted area plays a significant independent role in VDD and, hence, where they reside can potentially be one of the main influencers of vitamin D status in women. Season, time of day, cloud cover, smog, skin melanin content and sunscreens are among the factors that influence UV exposure and vitamin D synthesis [8]. Inadequate radiation or the insufficient cutaneous absorption of UVB is one of the cardinal causes of VDD [7]. There are a lot of studies that dealt with the seasonal variations of serum 25(OH)D and its correlation with UV radiation dose [9] but also with physical activity [10]. However, there are quite a few studies that claimed that the prevalence of VDD had no seasonal variation and remained stable, even in a sunny climate [9,11,12]. The results of the epidemiological study by Xyda et al. [13] demonstrate the burden of 25(OH)D deficiency in two sunny Mediterranean countries, Greece and Cyprus. They showed that the prevalence of VDD is extremely high in both population samples. The study by Dimakopoulos et al. [14] assessed the vitamin D status of the adult Greek population in relation to dietary intake, sun exposure and other factors, using data from the Hellenic National Nutrition and Health Survey (HNNHS). To our knowledge, this was the first study that aimed to identify factors associated with 25(OH)D concentration < 20 ng/mL in Greek adults. The HNNHS measurements showed that despite the abundant sun in Greece, 36% of adults had an insufficient concentration of 25(OH)D in serum (12–19.9 ng/mL), and 28.8% had an insufficient concentration, i.e., a concentration < 20 ng/mL. Many factors that could help improve vitamin D status were modifiable, including weight management, increased physical activity, skin exposure to sunlight and the use of supplements. Despite the study’s null findings between serum 25(OH)D concentration and dietary vitamin D intake, the latter was extremely low in almost the entire population, so they concluded that efforts to increase vitamin D intake are also required. Consequently, food fortification and vitamin D supplements are two options towards that goal. In addition, longer but safe sun exposure could offer an additional effective and low-cost strategy for VDD prevention.

In 2010, the Food and Nutrition Board at the Institute of Medicine of the National Academies established that an adequate intake of vitamin D during pregnancy was 600 international units (IU) per day [15]. Most prenatal vitamins typically contain 400 IU of vitamin D per tablet [15]. The authors of a recent clinical report from the Committee on Nutrition of the American Academy of Pediatrics suggested that a daily intake higher than that recommended by the Food and Nutrition Board may be needed to maintain maternal 25(OH)D sufficiency [15]. Although data on the safety of higher doses are lacking, most experts agree that supplemental vitamin D is safe in dosages up to 4000 IU per day during pregnancy or lactation. Recommendations concerning routine vitamin D supplementation during pregnancy beyond that contained in a prenatal vitamin should await the completion of ongoing randomized clinical trials [15]. The Greek National Nutrition Guide for pregnant and lactating women of the Institute of Preventive Environmental and Occupational Medicine warns about the need to take a vitamin D supplement during pregnancy; the WHO mentions the possibility of using a vitamin D supplement to adequately cover the intake of the above vitamin (FAO/WHO, 2004) [16].

The review by Harvey et al. [17] showed a modest positive association between maternal 25(OH)D levels and neonatal birth weight based on three observational studies. The study by Khalessi et al. [18] claimed that maternal VDD may increase the risk of having a low birth weight (LBW) neonate, and therefore, modifying maternal nutrition behavior and vitamin D levels could be beneficial to prevent LBW neonates. The study by Dalgard et al. [19] investigated whether insufficient cord 25(OH)D levels during early development could potentially negatively affect offspring development. They argued that umbilical cord 25(OH)D concentrations were positively related to neonatal length but not to birth weight and head circumference (HC). Neonates whose umbilical 25(OH)D concentrations were <12 nmol/L were 0.49 cm shorter than neonates with umbilical 25(OH)D levels > 50 nmol/L (95%CI: −0.85–−0.12). Another clinical study by Moradi et al. [20] showed that mothers of newborns with HC < 33 cm had significantly lower 25(OH)D levels compared to mothers of newborns with normal HC (*p* = 0.007). A cross-sectional study, similar to ours, by Viljakainen et al. [21] determined the association of mothers’ vitamin D status with bone variables of their newborns in order to assess whether maternal vitamin D status is prognostic to neonatal skeletal development. Their results suggested that maternal vitamin D status affected bone mineral accrual, such as bone mineral density (BMD), bone mineral content (BMC) and the cross-sectional area (CSA) of their fetus during the intrauterine period and influenced bone size of their neonates. Kilicaslan et al. [22], who also evaluated the association of maternal 25(OH)D levels with neonatal parameters at birth, argued that the height (*p* = 0.004), HC (*p* = 0.003) and chest circumference (*p* = 0.005) of newborns whose mothers received vitamin D supplementation was higher compared to mothers who did not. This appears to be explained by the fact that maternal 25(OH)D levels were significantly higher in pregnant women who received vitamin D supplementation during pregnancy (*p* < 0.001). The clinical study by Motamed et al. [4] evaluated the effectiveness of vitamin D supplementation of 1000 IU and 2000 IU in pregnancy in modulating maternal 25(OH)D levels, 25(OH)D concentrations in the umbilical cord, inflammatory biomarkers and adverse maternal–neonatal outcomes. Pregnant women receiving a 2000 IU vitamin D supplement gave birth to infants with significantly higher weight, length and HC compared to those infants whose mothers received a 1000 IU vitamin D supplement. However, in this study, maternal 25(OH)D was assessed not at birth but at the beginning of pregnancy and at 32 weeks of gestation. Vafaei et al. [3] evaluated the effect of low-dose vitamin D supplementation during pregnancy on fetal bone anthropometric aspects. They compared giving pregnant women vitamin D supplementation of either 1000 IU/day or placebo/day. What they found was that there was no statistically significant difference between the two doses in the cephalic length (CRL) (*p* = 0.93) and fetal thigh length (FL) (*p* = 0.54) of the newborns in the first trimester of pregnancy. However, there was a statistically significant difference in higher measures of these characteristics in the second and third trimesters of pregnancy (*p* < 0.001) in newborns whose mothers received a vitamin D supplement of 1000 IU compared with those whose mothers received a placebo.

In contrast to all of the above, Thompson et al. [23], who assessed the causal effects of maternal 25(OH)D levels and calcium supplementation on offspring birth weight in pregnancy, found no effect on birth weight in otherwise healthy neonates. The study by Almidani et al. [24] reached a similar conclusion; the association of neonatal birth weight with maternal 25(OH)D levels during pregnancy was statistically insignificant for both early term (after 37 + 0 to 38 + 6 weeks of gestation) and full-term infants (after 39 to 40 weeks of gestation). The clinical study by Hajhashemi et al. [25] tested the administration of a higher vitamin D dose supplement of 4000 IU/day for 10 weeks in pregnant women with VDD. They argued that although a higher dose supplement was clearly more beneficial than sun exposure (30 min daily on 30% of body surface area, in direct sunlight, summer, from 10 a.m. to 4 p.m.) in increasing maternal levels of 25(OH)D (*p* < 0.001), it did not appear to be associated with a difference in the height (*p* = 0.118), weight (*p* = 0.245) and HC (*p* = 0.681) of the newborn. The study by Gale et al. [26] suggested that exposure to higher maternal concentrations of 25(OH)D during pregnancy (>75 nmol/L) does not influence the neonate’s growth. It is worth mentioning that a clinical study by Aji et al. [27], based on its findings, may change the conditions in prenatal control after further research. The study by Aji et al. [27] examined the interaction between genetic variants involved in vitamin D synthesis and metabolism with maternal levels of 25(OH)D on neonatal anthropometric characteristics. This study showed that there was no significant relationship between maternal levels of 25(OH)D and anthropometric characteristics of the newborn (*p* > 0.05). However, when calculated and studied simultaneously, the genetic risk score (GRS) showed a statistically significant association with maternal levels of 25(OH)D in the third trimester of pregnancy (*p* = 0.004). And while the association of GRSs and anthropometric measurements did not exist alone, there was an interaction of GRSs and maternal 25(OH)D levels on neonatal HC (*p* = 0.030). Mothers of infants with an HC < 35 cm had significantly lower 25(OH)D levels if they carried >4 alleles compared to those who carried <3.

This observational study aims to evaluate any likely correlation between maternal VDD and neonatal anthropometrics (weight, height and HC) at birth while simultaneously evaluating the corresponding effects of vitamin D intake on this potential association by examining the samples of pregnant Greek women and their neonates, who benefited from the sun and Greece’s Mediterranean climate.

## 2. Materials and Methods

The present study is a cross-sectional, observational study that was conducted from September 2019 to January 2022 at the Tzaneio General Hospital of Piraeus. Criteria for participation in the present study were pregnant Greek women (≥37 weeks of gestation) giving birth at the Obstetrics and Gynecology clinic of the Tzaneio General Hospital of Piraeus who had a singleton live birth. Exclusion criteria were women taking medications that could potentially affect vitamin D levels (corticosteroids, anti-epileptics, anti-tuberculosis, anti-fungals) or those receiving higher doses of 25(OH)D supplementation (>800 IU). Pregnant women with a known medical history of rheumatoid arthritis, thyroid, parathyroid or adrenal disorders, liver or kidney failure, metabolic bone disease, type 1 DM and malabsorption (pancreatic failure, fibrocystic disease, celiac disease) were also excluded. This study was designed to describe if there is a possible association between maternal VDD and neonatal neonatal-specific anthropometric characteristics (weight, height and HC) at birth, taking into account the administration or not of prenatal vitamin D supplements. 

Data were collected from 248 healthy Greek mother–-newborn pairs at birth. We performed a study on 248 early-term infants (after 37 + 0 to 38 + 6 weeks of gestation), but also in full-term infants (after 39 + 0 to 40 weeks of gestation) and their Greek mothers in order to reveal any association between maternal VDD and neonatal specific anthropometric characteristics (weight, height and HC). The study was performed on maternal blood samples at the scheduled time of laboratory tests that are always performed before delivery and cord blood from the newborn immediately after ligation at the end of labor. A total of 5 mL of both mother’s and neonate’s cord blood were collected, labeled and sent to laboratory to measure 25(OH)D levels. The analysis of maternal 25(OH)D levels was a single test and was conducted only on the day of the delivery. We did not analyze maternal 25(OH)D levels at the beginning or throughout pregnancy. All collected blood samples were processed within the collection day. Both the maternal and neonatal blood samples were sent to the biochemistry laboratory of the Tzaneio General Hospital of Piraeus to measure 25(OH)D levels. We used a 25(OH)D ELISA kit, which was a complete kit for the quantitative determination of 25(OH)D in human plasma and serum samples. 

The sample size was initially determined to achieve its main objectives. The representativeness of the sample, not its size, is the most important measure and offers an increase in the degree of validity and reliability of a study [28]. More specifically, certain statistical rules were followed to ensure that the sample size was sufficient to perform the necessary statistical analyses that might show associations between maternal 25(OH)D levels and neonatal anthropometric characteristics. A sample of four hundred people would have been ideal; however, we settled for a sample of 250 people, not only because of the limited deliveries of our clinic but also because of the limited budget of the hospital and their subsequent inability to provide us with more than 500 kits to measure serum 25(OH)D. The evaluation of maternal/neonatal vitamin D concentrations was made according to the American Endocrine Society. All mothers were grouped according to 25(OH)D levels into those with (a) adequate levels of vitamin 25(OH)D (>30 ng/mL) [29], (b) a deficiency of vitamin 25(OH)D (21–29 ng/mL) [29], (c) a lack of vitamin 25(OH)D (<20 ng/mL) [29]. Here, perhaps there is another category (d) of severe vitamin deficiency 25(OH)D (<12 ng/mL) that could be added, given a review by Amrein et al. [30], which informed the current situation worldwide, regarding 25(OH)D and the risks arising from its severe lack, with a dramatic increase in the risk of mortality, infections but also many other diseases [30]. The newborns of the mothers of each category were also divided into newborns with (a) adequate levels of vitamin 25(OH)D (>30 ng/mL) [31], (b) vitamin 25(OH)D deficiency (16–29 ng/mL), (c) a lack of vitamin 25(OH)D (<15 ng/mL) [31], again according to the adequacy criteria of the American pediatric endocrine society. As with the adults, one more category could be added, (d) severe vitamin deficiency 25(OH)D (≤12.5 ng/mL) [32] or 25(OH)D (<10 ng/mL), given the most recent review of Bragger et al. [33] in which 25(OH)D levels in 248 maternal blood samples and cord blood levels of their newborns were studied. Maternal VDD was defined for values < 30 ng/mL, while values of ≥30 ng/mL were defined as adequate. Each mother’s results were accompanied by a detailed medical and personal questionnaire. Immediately after delivery, the somatometric characteristics of the newborn were collected. The recorded value of each neonatal’s somatometric characteristic was the average of three measurements taken either with a tape measure for the height and HC or with a well-calibrated scale for neonatal birth weight. Accurate measurement of birth weight required that neonates be weighed within one hour of birth. All measurements were made by midwives immediately after labor. The height of the neonate was measured from the top of their head to the bottom of one of their heels, while HC, the occipitofrontal circumference, was measured from above the supra-orbital ridge and around the occipital prominence. All other information related to the presence or absence of factors influencing the possible relationship of maternal VDD with neonate’s somatometric characteristics were drawn from a detailed medical history and a personal questionnaire that, as mentioned, accompanied each mother’s results. Maternal medical history included the presence or absence of GDM; preexisting diseases such as rheumatoid arthritis, thyroid, parathyroid or adrenal disorders, liver or kidney failure, metabolic bone disease, type 1 DM and malabsorption (pancreatic failure, fibro-cystic disease, celiac disease) that could potentially affect vitamin D levels; intake of any medication during and outside pregnancy; changes in maternal weight and body mass index (BMI) during pregnancy; intake or not of vitamin D supplements as well as possible complications of the present pregnancy. 

Investigation was carried out to understand if maternal VDD is an independent risk factor (RF) for reduced fetal–neonatal growth. Pregnant women received or did not receive a prenatal supplementation with a regular dose of calcium (500 mg/day) and vitamin D (400–800 IU/day), as instructed by their treating physicians. In our study, some of the doctors in our clinic administered vitamin D, and some did not. Under no circumstances were we able to modify this practice, which is in accordance with the guidelines of the Greek Ministry of Health that states that doctors may administer vitamin D supplements as required [16]. The only thing we did was to record which women took supplements. Immunological tests were used to measure maternal blood 25(OH)D levels. For this reason, we divided our sample into two groups: pregnant women who received vitamin D supplements and pregnant women who did not receive vitamin D supplements during pregnancy. After that, we examined the correlation of each of the somatometric characteristics with maternal VDD separately in each group. In order to find if there is a correlation, we used the Chi-square test to examine whether exposure to a risk factor (RF) leads to a particular outcome. We hypothesized that the RF is maternal VDD, and the outcome is either the birth of low birth weight (LBW) neonates or the birth of neonates with low height (<47 cm) or neonates with small HC (<33 cm). LBW neonates refer to neonates with birth weights < 2500 g. These neonates may be small for gestational age (GA) or have intrauterine growth restriction. Obviously, there are other factors besides maternal VDD that may have led to reduced somatometric characteristics, such as the presence of gestational diabetes mellitus (GDM), maternal BMI at the beginning of pregnancy, weight gain during pregnancy, the sex of the child, smoking, caffeine intake, physical exercise of the pregnant woman, maternal age, father’s height, socioeconomic status and neonatal’s 25(OH)D levels. All these factors were studied by multiple regression analysis to explain the statistical probability of each factor acting on the specific studied anthropometric characteristic.

Data were processed using IBM SPSS Statistics 26 software. Results of maternal and neonatal 25(OH)D levels are presented as means ± standard deviations (SD) or by frequencies and percentages. First of all, the study evaluated the mean maternal serum vitamin D level in all Greek mothers. As we said, depending on the mother’s 25(OH)D level, all mothers were categorized into adequate, deficient, severely deficient and lack of 25(OH)D. The Chi-square test was applied where applicable. We used Chi-square test to find out if maternal vitamin D intake during pregnancy (after 20 weeks of gestation) affects the somatometrics of the newborn (neonatal weight, height and head circumference). We then divided our sample into two groups of pregnant women who received vitamin D 400–800 IU and those who did not. We sought whether exposure of the newborn to maternal VDD results in lower body weight, smaller HC or smaller height of newborns in each group separately. We also used Chi-square test to determine if the factors from the above association are associated. Therefore, in the group where pregnant women were receiving vitamin D, we performed three different Chi-square tests for each one of the three somatometric characteristics of neonates to be examined, and similarly, three tests were examined, respectively, for each newborn somatometric characteristic in the group of mothers who did not receive vitamin D. As maternal VDD we defined 25(OH)D levels (<30 ng/mL) and as adequacy levels (≥30 ng/mL). We sought to find whether there were significant statistical differences between maternal VDD and neonates with height < 47 cm or neonates with height ≥ 47 cm, neonates with head circumference (HC) < 33 cm or neonates with HC ≥ 33 cm and neonates with birth weight < 2500 g and ≥2500 g. Multiple regression analysis was used to assess the statistical significance of other factors beyond maternal VDD and vitamin D intake that may also influence somatometric characteristics. These analyses were recorded in three separate tables. Each analysis had a different anthropometric characteristic (weight, height and head circumference) as a dependent variable. The independent, dichotomous variables we included in each table were the presence or absence of GDM, maternal BMI at the beginning of pregnancy (<25 or >25), weight gain during pregnancy (>14 kg or <14 kg), maternal vitamin D intake (yes, no), maternal 25(OH)D (>30 ng/mL or <30 ng/mL), neonatal 25(OH)D (>30 ng/mL or <30 ng/mL), gender of the child (male, female), smoking (yes, no), caffeine intake (yes, no), physical activity of the pregnant woman (increased, decreased), mother’s age (>35 years, <35 years), father’s height (>1.75 cm, <1.75cm) and economic status (good, poor). Maternal BMI was calculated from weight and height measurements recorded on their first and last visit to our hospital. According to the criteria of the WHO [34], normal BMI was (<25), overweight was (25–30) and obese was (>30). Pregnant women received or did not receive a regular dose of calcium (500 mg/day) and vitamin D (400–800 IU/day) supplementation, according to the instructions of their treating physicians. The personal questionnaire included maternal demographic and phenotypic characteristics, maternal dietary habits, smoking, caffeine intake, physical activity, educational level, socioeconomic status and husband’s height. The result of the analysis in each table could predict the probability of each independent variable influencing the respective anthropometric characteristics. To determine the statistical significance of each independent variable, the *p* value was considered. All *p*-values less than 0.05 (*p* ≤ 0.05) were defined as statistically significant. 

## 3. Results

Based on our sample, the percentage of clinical hypovitaminosis 25(OH)D, including a lack (<20 ng/mL) and severe deficiency (<12 ng/mL) of 25(OH)D, in mothers in Greece was found to be 58% (143/248). If we add the simple deficiency of 25(OH)D (<30 ng/mL), 25% (62/248), total maternal VDD increases to 83%, while only the remaining 17% of pregnant women samples exhibited 25(OH)D adequacy. Both maternal and neonatal 25(OH)D levels benefited from prenatal supplemental vitamin D intake. The mean of maternal and neonatal 25(OH)D concentrations in women who received vitamin D prenatally were 26.92 ± 12.43 ng/mL and 18.10 ± 8.24 ng/mL, respectively, vs. 16.92 ± 9.57 ng/mL and 12.64 ± 8.06 ng/mL in women and newborns who did not receive vitamin D. 

Our study revealed no association between maternal VDD and neonatal height or HC. More specifically, maternal VDD does not appear to be associated with a reduction in neonatal HC at birth. The association of maternal vitamin D status (VDD or not) with neonatal HC (<33 or ≥33) immediately after delivery is demonstrated in Table 1. In the group of mothers who received vitamin D supplementation during pregnancy (n = 83) and had maternal VDD, 16 neonates were born with HC < 33 cm compared with 41 cases with HC ≥ 33 cm. A Chi-square test analysis gave a *p*-value (*p* = 0.172) > 0.05%. In the group of mothers who did not receive vitamin D supplementation during pregnancy (n = 165) and had maternal VDD, 40 neonates were born with HC < 33 cm compared with 108 cases with HC ≥ 33 cm. The Chi-square test analysis gave a *p*-value (*p* = 1) > 0.05%. Based on the *p* values of the Chi-square tests, we could not reject the null hypotheses, and therefore, we accepted that maternal VDD does not lead to a reduced HC of newborns for both groups. Similarly, maternal VDD does not appear to be associated with reduced height at birth. The association of maternal vitamin D status (VDD or not) with neonatal height (<47 cm or ≥47 cm) immediately after delivery is shown in Table 2. In the group of mothers who received vitamin D supplementation during pregnancy (n = 83) and had maternal VDD, 6 neonates were born with reduced height < 47 cm compared with 51 cases with neonatal height ≥ 47 cm. A Chi-square test analysis gave a *p*-value (*p* = 0.462) > 0.05%. Thus, we could not reject the null hypothesis and, therefore, accepted that maternal VDD does not lead to reduced neonatal height at birth in infants of mothers who received vitamin D supplements during pregnancy. In the group of mothers who did not receive vitamin D supplementation during pregnancy (n = 165) and had maternal VDD, 15 neonates were born with neonatal height < 47 cm compared with 133 cases with HC ≥ 47 cm. A Chi-square test analysis gave a *p*-value (*p* = 0.002) < 0.05%. That being the case, we initially rejected the null hypothesis and, therefore, accepted that maternal VDD leads to a reduced height in newborns at birth whose mothers did not receive vitamin D supplements during pregnancy. However, because the Odds Ratio was 1.133 and the 95% Confidence Interval (CI) was between 0.953 to 1.348, which included the value 1, we remained in the null hypothesis that there is a high probability that maternal VDD is not related to reduced height in newborns at birth whose mothers did not receive vitamin D supplements during pregnancy. 

On the other hand, our study revealed a statistically significant relationship between maternal VDD and LBW neonates (Table 3). In our study, our findings demonstrated a positive association between maternal VDD and LBW neonates whose mothers received vitamin D during pregnancy. In the group of mothers who received vitamin D supplementation during pregnancy (n = 83) and had maternal VDD, 4 LBW neonates were born compared with 53 cases with normal birth weight ≥ 2500 g. A Chi-square test analysis gave a *p*-value (*p* = 0.001) < 0.05%. As a result, we rejected the null hypothesis and accepted that maternal VDD leads to LBW newborns whose mothers received vitamin D supplements during pregnancy. In the group of mothers who did not receive vitamin D supplementation during pregnancy (n = 165) and had maternal VDD, 0 newborns were born with a newborn weight < 2500 g compared with 17 cases with a newborn weight ≥ 2500 g. The Chi-square test analysis gave a *p*-value (*p* = 0.352) > 0.05%. Therefore, we accepted that maternal VDD is not related to the birth weight of newborns born to mothers who did not receive vitamin D supplements during pregnancy.

Although pregnant women’s intake of vitamin D supplements during pregnancy increased their serum 25(OH)D levels, it was statistically significantly associated only with neonatal birth weight and not with neonatal birth height or HC. Through multiple regression analysis, we revealed the statistical possibility of factors other than maternal vitamin D intake that may be associated with neonatal anthropometric characteristics. Neonatal HC appeared to be related to initial pregnancy weight (*p* = 0.032), maternal weight gain (*p* = 0.045), husband’s height (*p* = 0.025), child’s gender (*p* = 0.000) and mother’s activity (*p* = 0.007) (Table 4). Neonatal weight was statistically significantly associated with GDM (*p* = 0.016), child’s gender (*p* = 0.002) and maternal weight at the beginning of pregnancy (*p* = 0.013) (Table 5). Finally, neonatal height was statistically significantly associated with GDM (*p* = 0.02) and neonatal sex (0.006) (Table 6).

## 4. Discussion

Few studies evaluate maternal VDD with neonatal anthropometrics such as weight, height and HC in Mediterranean countries. This research was designed to investigate the maternal status of VDD at the time of delivery in healthy pregnant Greek mothers and to document whether this is related to the somatometric characteristics of their newborns. We also examined whether the above relationship is likely to be affected by prenatal vitamin D supplementation. In order to demonstrate the role of maternal VDD in the somatometric characteristics of newborns at birth, prospective clinical studies are definitely required to support or reject any conclusions of our research. If a correlation is proven between maternal VDD and neonatal somatometric characteristics, it would be of interest to develop a prenatal pregnancy selection program which will monitor and accordingly adjust VDD early or during pregnancy in order to improve fetal–neonatal development. Given the benefit of sunlight in vitamin D synthesis in a Mediterranean country like Greece, such a program would enable health professionals to correct the levels of vitamin D in pregnant women, if deemed necessary, through the recommendation of more appropriate vitamin D supplements. Contrary to expectations, a high prevalence of maternal VDD was seen in Greek women at birth. Furthermore, maternal VDD at birth did not appear to be related to the offspring’s neonatal height and HC but probably only to their neonatal weight, measured immediately after birth.

Due to the nature of the study, some limitations should be considered. Because our study is a cross-sectional one, no causal relationships can be drawn. One of the main drawbacks of our study was a relatively small sample size. A sample of four hundred people would be ideal; however, we settled for a sample of 250 people, not only because of the limited deliveries at our clinic but also because of the limited hospital budget and their subsequent inability to provide us with more than 500 kits for measuring serum 25(OH)D. According to Gorsuch et al. [35], a sample rule of one hundred individuals is considered necessary to perform factorial statistical analyses. Our sample selection may not be ideal, but it seems consistent with the sample size used by previous cross-sectional studies in a similar research field and topic, such as the study by Khalessi et al. [18] that was performed on 100 pregnant women at term and on 100 newborns born to these mothers, the study by Viljakainen et al. [21] that was performed on 125 pregnant women and their neonates and the study by Kilicaslan et al. [22] that was performed on 100 pregnant women and their 100 newborns. But because, according to McNutt [36], the sample size of a study depends on the incidence of the disease in the unexposed population, and because the incidence of VDD is considered quite high, our sample is again considered too small to answer the research questions, reduce errors and draw safe statistical conclusions, which can be generalized to the general population [37,38]. Furthermore, we did not measure maternal PTH, calcium and phosphorus values, which are related to 25(OH)D levels. Several other limitations that might have biased our results was the fact that the analysis of maternal 25(OH)D levels was a single test and was not conducted at the start or during pregnancy but only on the day of delivery and, as such, does not imply that the recorded levels are typical for the entire period of pregnancy. For example, other studies, such as that of Miliku et al. [39], examined whether maternal 25(OH)D levels in the second trimester of pregnancy influenced fetal growth patterns and birth outcomes. Indeed, they suggested that when maternal 25(OH)D levels were low in the second trimester, fetuses had smaller HC, length and birth weight (*p* < 0.05) in the third trimester. In contrast, in our study, we found no relevant differences between maternal 25(OH)D levels and neonatal HC or neonatal height at birth, but we found a statistically significant association of maternal VDD with neonatal weight in measurements made at the end of pregnancy (for maternal 25(OH)D levels) and immediately after birth (for neonatal 25(OH)D levels). If more than one measurement of the maternal status of 25(OH)D was performed during pregnancy and not only at the time of delivery, a statistically significant association of maternal VDD with the other two neonatal characteristics (height and HC) under examination might have emerged. Another shortcoming of our study is that we did not perform a nutritional assessment of the pregnant mothers, as diet is one of the determinants of the condition of 25(OH)D levels. In addition, our doctors used low doses of (400–800 IU) vitamin D supplements. Although, in our study, we were able to find an association of maternal VDD at delivery only with neonatal birth weight, our finding seemed compatible with the study of Kilicaslan et al. [22], who also evaluated the association of maternal 25(OH)D levels with neonatal parameters at birth and argued that height, HC and chest circumference of newborns whose mothers received vitamin D supplementation were higher compared to mothers who did not. This appears to be explained by the fact that maternal 25(OH)D levels were significantly higher in pregnant women who received vitamin D supplementation during pregnancy. It is likely that the ideal administered dosage of vitamin D and the correct timing of its initiation in pregnancy may have an important impact on newborn somatometry. Ιf we had administered a higher dose of vitamin D supplementation, this could possibly have engendered changes in the rest of the somatometric characteristics of the newborn we were studying, as well as in further neonatal development. However, even Vaziri et al. [40], who evaluated the effect of vitamin D supplementation of 2000 IU in the mother–newborn pair in late pregnancy (at 26–28 weeks of gestation until delivery), did not find any effect on somatometric measurements of the newborns and in the bone mass improvement of the newborn. The review by Harvey et al. [17] showed a modest positive association between maternal 25(OH)D levels and neonatal birth weight based on three observational studies. Although there was modest evidence to support a relationship between maternal 25(OH)D levels and offspring birth weight, bone mass and serum calcium concentrations, these findings were limited by their observational nature (birth weight, bone mass) or the risk of bias and the low quality of the studies (calcium concentrations). Therefore, caution is advised when formulating final conclusions or generalizing our results. 

## 5. Conclusions

Our study highlighted that maternal VDD in healthy pregnant Greek women at birth does not appear to play a statistically significant role in all the anthropometric characteristics we studied except for neonatal birth weight. We demonstrated a direct correlation between maternal VDD at the end of gestation and LBW neonates, indicating a positive connection between neonatal birth weight and serum 25(OH)D levels in mothers who received vitamin D supplementation. It was considered plausible that this occurred because vitamin D supplementation increased maternal 25(OH)D levels. Consequently, we observed that a possible correction of maternal 25(OH)D levels could prevent impaired neonatal growth. In any case, further prospective studies are needed to further clarify the specifics of the role of maternal VDD on neonatal somatometric characteristics and to determine the adequate values of 25(OH)D concentrations required during pregnancy, taking into account other factors that seem to influence fetal–neonatal growth. In such studies, the frequency, ideal dose and time of sampling play a decisive role, as well as increased sunshine. 

## Figures and Tables

**Table 1:** Correlation of maternal VDD with neonatal head circumference (HC) in two different groups of mothers who took vitamin D supplements in pregnancy and mothers who did not.

**Group of Mothers Who Received Vitamin D Supplements**					
**Chi-Square Tests**					
	**Value**	**df**	**Asymptotic Significance** **(2-Sided)**	**Exact Sig.** **(2-Sided)**	**Exact Sig.** **(1-Sided)**
Pearson Chi-Square	2.612	1	0.106		
Continuity Correction	1.866	1	0.172		
Likelihood Ratio	2.556	1	0.110		
Fisher’s Exact Test				0.135	0.087
Linear-by-Linear Association	2.581	1	0.108		
No. of Valid Cases	83				
**Group of Mothers Who Did Not Receive Vitamin D Supplements**					
**Chi-Square Tests**					
	**Value**	**df**	**Asymptotic Significance** **(2-Sided)**	**Exact Sig.** **(2-Sided)**	**Exact Sig.** **(1-Sided)**
Pearson Chi-Square	0.044	1	0.834		
Continuity Correction	0.000	1	1.000		
Likelihood Ratio	0.043	1	0.836		
Fisher’s Exact Test				0.781	0.517
Linear-by-Linear Association	0.043	1	0.835		
No. of Valid Cases	165				

**Table 2:** Correlation of maternal VDD with neonatal height in two different groups of mothers who took vitamin D supplements in pregnancy and mothers who did not.

**Group of Mothers Who Received Vitamin D Supplements**					
**Chi-Square Tests**					
	**Value**	**df**	**Asymptotic Significance** **(2-Sided)**	**Exact Sig.** **(2-Sided)**	**Exact Sig.** **(1-Sided)**
Pearson Chi-Square	1.177	1	0.278		
Continuity Correction	0.541	1	0.462		
Likelihood Ratio	1.117	1	0.291		
Fisher’s Exact Test				0.308	0.227
Linear-by-Linear Association	1.163	1	0.281		
No. of Valid Cases	83				
**Group of Mothers Who Did Not Receive Vitamin D Supplements**					
**Chi-Square Tests**					
	**Value**	**df**	**Asymptotic Significance** **(2-Sided)**	**Exact Sig.** **(2-Sided)**	**Exact Sig.** **(1-Sided)**
Pearson Chi-Square	17.625	1	0.000		
Continuity Correction	9.170	1	0.002		
Likelihood Ratio	9.312	1	0.002		
Fisher’s Exact Test				0.010	0.010
Linear-by-Linear Association	17.519	1	0.000		
No. of Valid Cases	165				

**Table 3:** Correlation of maternal VDD with neonatal weight in two different groups of mothers who took vitamin D supplements in pregnancy and mothers who did not.

**Group of Mothers Who Received Vitamin D Supplements**					
**Chi-Square Tests**					
	**Value**	**df**	**Asymptotic** **Significance (2-Sided)**	**Exact Sig.** **(2-Sided)**	**Exact Sig.** **(1-Sided)**
Pearson Chi-Square	14.179	1	0.000		
Continuity Correction	10.947	1	0.001		
Likelihood Ratio	14.990	1	0.000		
Fisher’s Exact Test				0.001	0.001
Linear-by-Linear Association	14.008	1	0.000		
No. of Valid Cases	83				
**Group of Mothers Who Did Not Receive Vitamin D Supplements**					
**Chi-Square Tests**					
	**Value**	**df**	**Asymptotic Significance** **(2-Sided)**	**Exact Sig.** **(2-Sided)**	**Exact Sig.** **(1-Sided)**
Pearson Chi-Square	1.895	1	0.169		
Continuity Correction	0.867	1	0.352		
Likelihood Ratio	3.429	1	0.064		
Fisher’s Exact Test				0.370	0.181
Linear-by-Linear Association	1.884	1	0.170		
No. of Valid Cases	165				

**Table 4:** The descriptive statistics of head circumference (HC) with selected parameters.

Dependent Variable: Head Circumference (HC)					
	Unstandardized Coefficients		Standardized Coefficients	t	Sig.
	B	Std. Error	Beta		
Independent variables (Constant)	33.574	0.748		44.885	0.000
GDM	0.335	0.298	0.068	1.126	0.261
BMI beginning of pregnancy	−0.058	0.038	−0.237	−1.501	0.135
maternal D	−0.255	0.283	−0.059	−0.903	0.368
neonatal D	−0.100	0.467	−0.013	−0.214	0.831
husband height	0.006	0.003	0.139	2.249	0.025
maternal age	0.120	0.195	0.037	0.616	0.538
weight gain in pregnancy	0.006	0.003	0.122	2.016	0.045
initial pregnancy weight	0.029	0.014	0.340	2.163	0.032
financial situation	−0.233	0.219	−0.069	−1.068	0.287
smoking	0.338	0.213	0.103	1.589	0.114
mother’s activity	−0.975	0.361	−0.170	−2.705	0.007
child gender	−0.797	0.195	−0.248	−4.088	0.000
vitamin D intake	−0.033	0.213	−0.010	−0.154	0.878
caffeine	−0.315	0.210	−0.098	−1.503	0.134

The statistical significance is indicated in the table as positive when the *p*-value ≤ 0.05.

**Table 5:** The descriptive statistics of neonatal weight with selected parameters.

Dependent Variable: Neonatal Weight					
	Unstandardized Coefficients		Standardized Coefficients	t	Sig.
	B	Std. Error	Beta		
Independent Variables (Constant)	2986.019	267.694		11.155	0.000
GDM	259.427	106.671	0.152	2.432	0.016
BMI beginning of pregnancy	−23.166	13.767	−0.273	−1.683	0.094
maternal D	−21.490	101.254	−0.014	−0.212	0.832
neonatal D	65.106	167.211	0.025	0.389	0.697
husband height	0.954	1.003	0.061	0.951	0.342
maternal age	54.149	69.956	0.048	0.774	0.440
weight gain in pregnancy	0.876	1.091	0.050	0.803	0.423
initial pregnancy weight	12.207	4.862	0.406	2.511	0.013
financial situation	−136.430	78.236	−0.115	−1.744	0.083
smoking	44.758	76.106	0.039	0.588	0.557
mother’s activity	−227.807	129.021	−0.114	−1.766	0.079
child gender	−214.013	69.779	−0.191	−3.067	0.002
vitamin D intake	109.121	76.250	0.096	1.431	0.154
caffeine	−61.784	75.049	−0.055	−0.823	0.411

The statistical significance is indicated in the table as positive when the *p*-value ≤ 0.05.

**Table 6:** The descriptive statistics of neonatal height with selected parameters.

Dependent Variable: Neonatal_Height					
	Unstandardized Coefficients		Standardized Coefficients	t	Sig.
	B	Std. Error	Beta		
Independent Variables (Constant)	51.456	2.136		24.088	0.000
GDM	−1.067	0.830	−0.081	−1.285	0.020
weight gain in pregnancy	0.009	0.009	0.063	1.007	0.315
BMI beginning of pregnancy	0.015	0.043	0.022	0.343	0.732
maternal vitamin D	0.021	0.040	0.057	0.528	0.598
neonatal vitamin D	−0.013	0.053	−0.025	−0.239	0.812
financial situation	0.117	0.607	0.013	0.193	0.847
husband height	0.002	0.008	0.015	0.232	0.817
smoking	0.285	0.598	0.032	0.476	0.634
vitamin D intake	−0.077	0.613	−0.009	−0.126	0.900
mother’s activity	−3.962	1.004	−0.257	−3.948	0.080
child gender	−1.503	0.542	−0.174	−2.772	0.006
caffeine	−0.125	0.575	−0.014	−0.218	0.828
maternal age	0.576	0.546	0.067	1.055	0.293

The statistical significance is indicated in the table as positive when the *p*-value ≤ 0.05.

## Data Availability

The data are not publicly available due to the Principle of Personal Data protection regulations but can be obtained upon a reasonable request to the corresponding author. Application number of Request to collect data: 7380/27 May 2019.
